# Silicon Carbide Technology for Advanced Human Healthcare Applications

**DOI:** 10.3390/mi13030346

**Published:** 2022-02-22

**Authors:** Stephen E. Saddow

**Affiliations:** 1 Electrical Engineering Department, University of South Florida, Tampa, FL 33620, USA; saddow@usf.edu; Tel.: +1-813-974-4773; 2 Department of Medical Engineering, University of South Florida, Tampa, FL 33620, USA

**Keywords:** silicon carbide, neural interface, biosensor, nanotechnology, MRI compatibility

## Abstract

Silicon carbide (SiC) is a highly robust semiconductor material that has the potential to revolutionize implantable medical devices for human healthcare, such as biosensors and neuro-implants, to enable advanced biomedical therapeutic applications for humans. SiC is both bio and hemocompatible, and is already commercially used for long-term human in vivo applications ranging from heart stent coatings and dental implants to short-term diagnostic applications involving neural implants and sensors. One challenge facing the medical community today is the lack of biocompatible materials which are inherently smart or, in other words, capable of electronic functionality. Such devices are currently implemented using silicon technology, which either has to be hermetically sealed so it does not directly interact with biological tissue or has a short lifetime due to instabilities in vivo. Long-term, permanently implanted devices such as glucose sensors, neural interfaces, smart bone and organ implants, etc., require a more robust material that does not degrade over time and is not recognized and rejected as a foreign object by the inflammatory response. SiC has displayed these exceptional material properties, which opens up a whole new host of applications and allows for the development of many advanced biomedical devices never before possible for long-term use in vivo. This paper is a review of the state-of-the art and discusses cutting-edge device applications where SiC medical devices are poised to translate to the commercial marketplace.

## 1. Introduction

Silicon carbide (SiC) has a long history as a robust and extremely hard material. First used as a cutting material in the 19th century and later as a high-temperature semiconductor for advanced applications in the 20th century, the history of SiC is quite interesting and the reader is referred to the first chapter in [1] which is dedicated to this subject. It is recommended that the reader also consult the literature to fully understand all of the many aspects of SiC: from how it is manufactured; the various crystal polytypes which each have slightly different physical, optical, and electrical properties; and, finally, the large number and types of applications it has been applied to. Fortunately, after a brief overview of SiC, the reader can understand why this material is so compelling for biomedical applications and why it may become one of the most used active biomaterials in the 21st century. Indeed, the purpose of this paper is to introduce SiC to biomedical engineers, scientists, and medical professionals, thus bringing together technologists from across many disciplines to stimulate the development and adoption of next-generation smart biomedical devices for advanced human healthcare applications. While this review refers primarily to the work of the author’s team, as they have been one of the principal pioneers in the development of advanced SiC devices for healthcare, it also will demonstrate that the field is growing and that other researchers have been looking at this material for advanced biomedical applications.

## 2. Biological Performance of SiC

SiC has a long history as a chemically robust material. Indeed, the inability to etch SiC using wet chemistry has made the fabrication of devices more challenging and expensive. However, it is this inherent ‘robust chemical resistivity’ material property that makes SiC attractive for in vivo biomedical applications. The human body is often referred to as a harsh environment. Any device interacting within this environment is constantly exposed to a salinated, ionic environment full of proteins which “foul” or coat it’s surface. Additionally, the body’s own active inflammatory system rapidly identifies foreign objects and activates to unleash various physical and chemical attacks, complete with oxidizers, in order to eliminate or dissolve the foreign intruder. Failure to eliminate the intruder will lead to the ‘walling off’ or otherwise separating of the device, shutting off its access to the bodily environment. A biomedical device fabricated from materials that do not succumb to inflammatory attacks and does not telegraph its presence by shedding ions to further alert the immune system is key to overall device functionality. The robust chemical nature of SiC has demonstrated, through many examples, minimal to unmeasurable immune system response when applied in chronic implantations [2,3]. In this section, we discuss the materials-level interaction of SiC and present some compelling work that shows that not only does SiC display excellent levels of compatibility with biological tissue but that it may be functionalized to further enhance its interaction, which is key to realizing in vivo biosensors.

### 2.1. In Vitro Performance

Cell-semiconductor hybrid systems are an important component of many biotechnological applications [4]. In this section, crystalline SiC is introduced as an extremely appealing semiconductor material for bio-applications. For the first time, an in vitro biocompatibility study of the main three SiC polytypes was conducted by Coletti et al. [4]. The reported results document the in vitro biocompatibility of all principal SiC polytypes and their capability of directly interfacing cells without the need of specific surface functionalization. SiC polytypism and doping concentration were found to have no influence on cell proliferation for the cell lines studied. Moreover, this study reported that SiC surfaces are a better substrate for mammalian cell cultures than Si in terms of both cell adhesion and proliferation. In the past, the fact that cells could be directly cultured on Si crystalline substrates led to the widespread use of this smart material in biotechnology [5,6,7]. The results reported in [2] define SiC as a promising substrate for future cell-semiconductor hybrid systems as a certain degree of bioactivity was observed for SiC when interacting with connective tissue cells. The main factors that have been shown to define SiC biocompatibility are its excellent tribological properties, hydrophilicity, and surface chemistry. SiC surface roughness variations within the nanometer scale have been shown to have minimal to no measureable effect on cell adhesion or proliferation. Both hydrofluric acid treatment, followed by a thorough deionized water rinse, and hydrogen annealing were shown to be suitable last steps to prepare SiC surfaces prior to cell plating. The extremely promising results reported by C. Coletti in [4] make SiC a promising material for biomedical devices ISO 10993 testing, which include the battery of tests closely monitored by the FDA for evaluating device acceptability for clinical trials. Indeed, amorphous SiC (*a*-SiC) and 3C-SiC were compared to other materials as part of a DARPA program to evaluate novel materials for neural probe applications, the details of which may be found in references [2,3].

**Figure 1:**
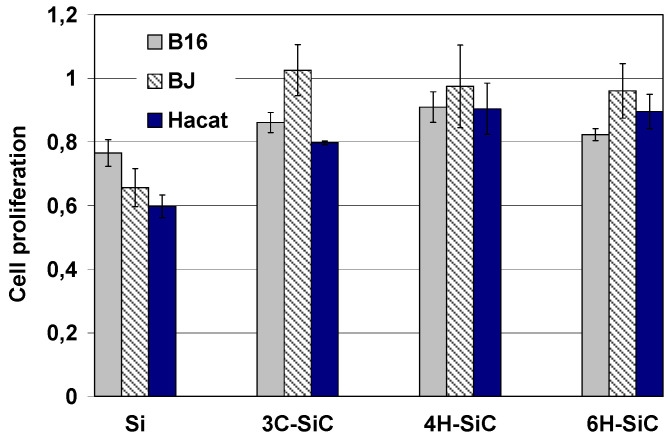
Cell proliferation of B16, BJ, and HaCaT cells expressed as $\bar{x}$ ± σ_m_ measured via MTT assays at the third day in vitro. Cell proliferation is greater on SiC than on Si surfaces for all the cell lines studied [4].

Numerous cell lines were studied to better understand competing literature reports that suggested that SiC was both bio-permissive and cytotoxic. By studying the main crystal forms of SiC, the apparent contradiction turned out to be likely due to nuances in how SiC should be prepared for cell culture. In the final analysis, SiC and its numerous polytypes and forms (amorphous, polycrystalline, monocrystalline, nano-structured, etc.) have all been shown to provide an excellent interface to the biological world (see Figure 1) [2,3].

**Figure 2:**
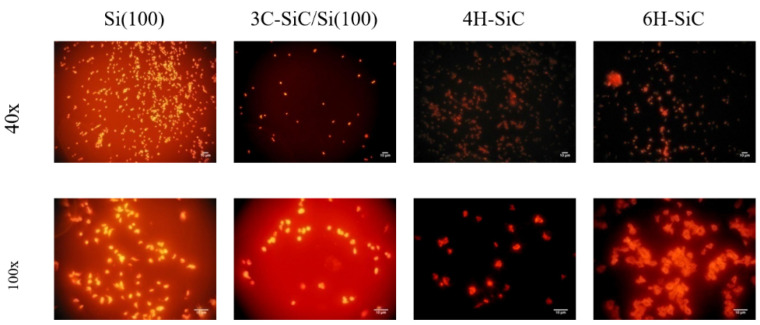
Static hemocompatibility study. Fluorescent micrographs comparing adhered platelets on three different SiC polytypes and Si. 3C-SiC clearly shows lower adhesion and high circularity while Si presented the highest adhesion. 4H and 6H-SiC evidenced the presence of clumps and a high degree of activation [2].

One of the important properties of implantable biodevices is hemocompatibility. This is particularly true for neural implants due to the dense vasculature in the brain. Two studies were conducted to assess hemocompatibility, namely the interaction of platelet-rich plasma (PRP) with various SiC and control surfaces in both static [2] and dynamic [3] modes. In the static study, it was observed that 3C-SiC produced little thrombotic reactivity, especially compared to Si when contacted with blood platelets, which was not surprising as Si is known to stimulate the clotting cascade (i.e., thrombosis). Interestingly, the hexagonal polytypes tested (4H and 6H-SiC) were no better than Si when in contact with PRP (see Figure 2) [2].

**Figure 3:**
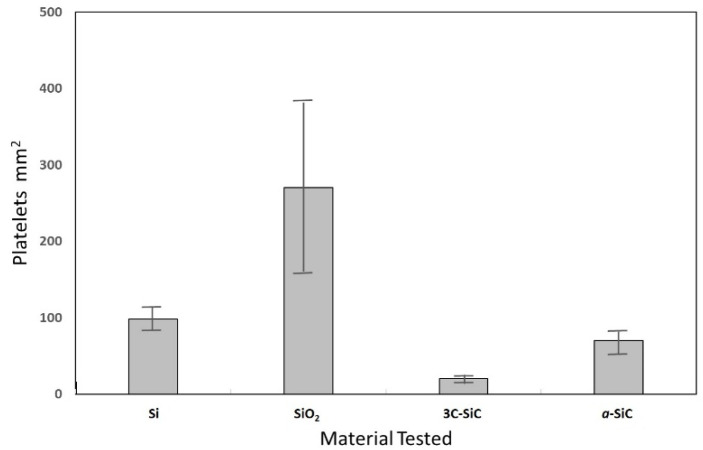
Dynamic hemocompatibility histogram of platelet activation of Si and SiO_2_ on Si and 3C-SiC, as well as *a*-SiC on Si, and using standard deviation as the error bar. Activated platelets per mm^2^ were used to evaluate the hemocompatibility of the materials. Test coupons: 50 × 75 mm^2^ [3].

Amorphous silicon carbide (*a*-SiC), which is an insulating material that can be used for biomedical devices, was not tested in the initial experiments, which were conducted under static conditions. Consequently, Nezifati then examined dynamic thrombotic conditons using a flowing Chandler loop to mimic the cardiovascular system [3]. Figure 3 shows a histogram comparing platelet adhesion to various surfaces and clearly demonstrates low platelet activity by 3C-SiC, especially compared to Si and SiO_2_. Interestingly, *a*-SiC only displayed a slightly lower platelet reaction when compared to Si under dynamic conditions.

### 2.2. In Vivo Performance

**Figure 4:**
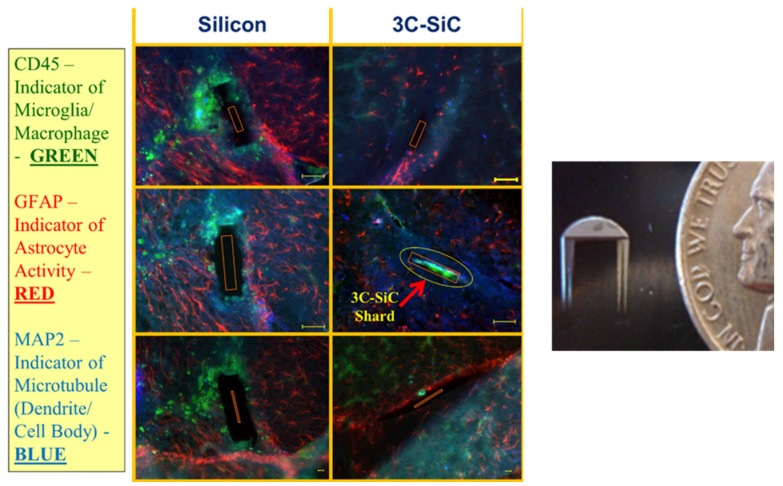
Microscope which was used for the immunofluorescence analysis of C57BL/6J mouse hippocampal tissue. Each row represents a selected tissue slice from a single animal (N = 3). The first column shows the tissue response within the left hemisphere of the hippocampus surrounding the Si implant and the second column presents the tissue associated with 3C-SiC within the right hemisphere. The legend on the left details the antibody stains used in the study. The orange boxes denote the approximate size of the implant at the tissue location. The scale bars are all 50 µm in length. Image from [3]. Right photograph of implanted passive probes showing Si (**left**) and 3C-SiC (**right**) double-shanks glued together for simultaneous implantation into wild-type mice. Red rectangles marking the implant dimension have been superimposed for reference, which show significant neural tissue loss extending beyond the Si implants while no tissue loss was observed for 3C-SiC implants.

Several in vivo studies were performed at the University of South Florida which served as further motivation for the development of SiC biomedical devices. Three animal models were studied (mouse, rat, and pig) and, in each case, a null immune system response to 3C-SiC and a-SiC was observed, whereas significant stimulation of the immune system was observed for Si. The first involved non-functional double-shank probes with Si on one side and 3C-SiC on the opposite (Figure 4), which were implanted into C57BL/6J wild-type mice for 35 days. For the first time, a comprehensive histological analysis of 3C-SiC for neural device applications was performed and reported in [3].

Perhaps the most important outcome of this work was the in vivo tissue comparison between identical form-fit Si and 3C-SiC passive double-shank devices. After harvesting tissue slices, the Si and 3C-SiC implant site were compared on the same slice from three different mice, with the resulting tissue histology data displayed in Figure 4.

A number of important observations can be made from the images related to the implant location. First, the left and right images are from the same tissue slice and show the areas where the Si and 3C-SiC implants were located, thus allowing for a meaningful direct comparison between the two materials within the same animal. A large area devoid of neural tissue is apparent around all of the Si implants. Additionally, the voids are often larger than 50 µm wide, which is a distance much larger than the implant thickness of ~20 µm. The voids for the areas where the 3C-SiC implant was removed are much smaller in comparison and closer to the original size of the implant for animals 1 and 2, which suggested that less overall damage has occurred to the brain. Tissue from the third animal was an exception, with a void width larger than 50 µm. While GFAP+ and CD45+ activity is increased in the areas of the implants for Si, 3C shows little appreciable activation. The third animal was the only animal which showed signs of increased GFAP+ activity in relation to 3C-SiC. Based on this work, functional SiC neural implants have been developed and are presented later in this paper and in [1].

**Figure 5:**
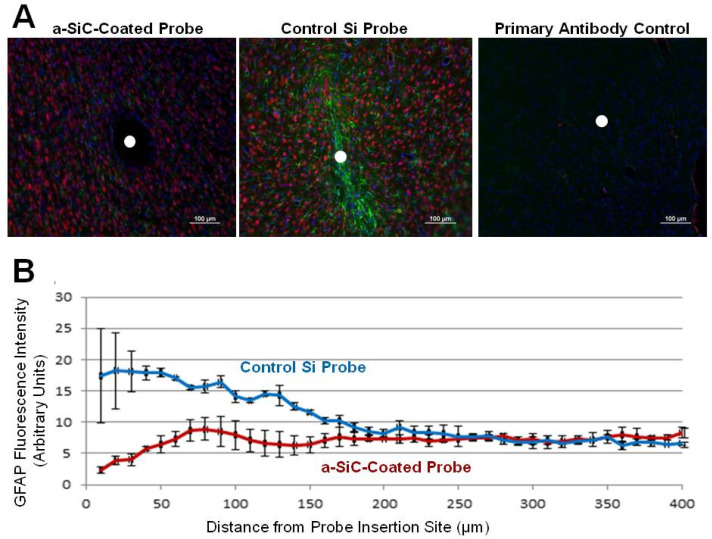
(**A**) Images from deep cortical tissue labeling NeuN in red (neurons), GFAP in green (astrocytes), and DAPI in blue (cell nuclei). White circles indicate center of probe locations. Left image is from tissue implanted with an a-SiC-coated probe, middle from a control Si probe, and the right image is the no primary control, indicating little or no non-specific antibody binding. (**B**) Immunohistochemistry summary data comparing results from a-SiC-coated (red) and control Si (blue) probe after implantation for four weeks. Data are mean ± SEM for n = 2 slices [3].

While these studies suggest that fully monolithic “All-SiC” biomedical devices or, in other words, devices composed completely of SiC materials may be homogeneously integrated for long-term human use, having clear advantages over Si or metal-based devices, SiC may be beneficial for other devices as well. In many neuroscience applications, long-term implantation is not a major issue and simply coating contemporary neural interfaces with a-SiC insulation provides numerous benefits. The work done by Pancrazio et al. has shown a significant in vivo improvement of SiC-coated implants, as shown in Figure 5.

## 3. SiC Biosensors

Numerous approaches to biosensing are available in the literature and is simply too exhaustive of a list to provide here. An overview of SiC biosensing that encompasses what is provided in this section may be found in [8]. Common to many biosensors is the need to functionalize the sensor surface to achieve adequate specificity (i.e., discrimination between target analyte and other components in the biosensor environment). Thus, we begin this section with a review of SiC surface functionalization followed by glucose and myoglobin sensing research. While this is not a complete review of all biosensor work in SiC, the objective is to provide some excellent examples of this important topic.

### 3.1. Surface Functionalization

In recent years, striking advancements in SiC growth and processing have enabled the deployment of this wide bandgap semiconductor in high-temperature, high-power, and harsh-environment applications. While important work towards improved solid-state devices continues, one of the most promising new frontiers for SiC lies at the inorganic/(bio)organic interface. Although the technological maturity of silicon has made it an obvious choice for biosensors and implantable electrodes, it lacks the chemical stability required for long-term in vivo operation and silicon dioxide is susceptible to the in-diffusion of ions (Na, K, and Fe), which can result in significant electronic drift currents and high levels of electronic noise. On the other hand, the chemical resistance, mechanical robustness, and biocompatibility of SiC are extremely favorable to long-term biological sensor applications.

**Figure 6:**
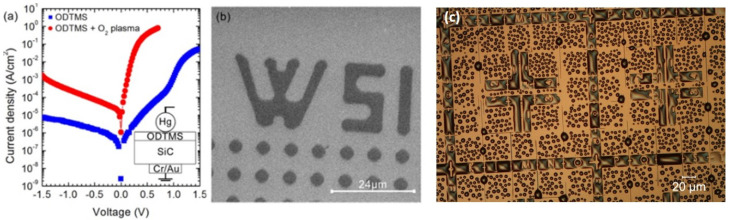
SAM synthesis (ODTMS-functionalized) on 6H-SiC. (**a**) IV curves and (**b**) SEM micrograph of regions with different electrical conductivities. Dark areas indicate oxygen plasma-treated regions. (**c**) Wettability image of micropatterned ODTMS-modified SiC surface. Note water droplets condense preferentially in hydrophilic oxygen plasma-exposed regions [2].

A natural starting point for the development of functional silicon carbide for biomedical and biosensing applications is its surface termination, which defines the chemical reactivity, surface defect density, work function, and ambient stability of the material. SiC surfaces have demonstrated excellent ability to host a wide range of organic molecules which have been covalently bound. These molecules possess specific head and end-groups which have been defined for chemical binding, allowing for interaction with the surrounding environment to graft biomolecules, such as specific proteins and DNA. In [2], some examples of self-assembled monolayer (SAM) formations with organosilanes and short-chain alkenes, as well as direct surface photopolymerization using vinyl monomers, were reported. In fact, a case example of how SAM-modified surfaces can be used to control the spatial wettability of silicon carbide for biomedical applications was provided and this work is shown below in Figure 6.

#### 3.1.1. SiC Functionalization to Enhance Cell Viability

**Figure 7:**
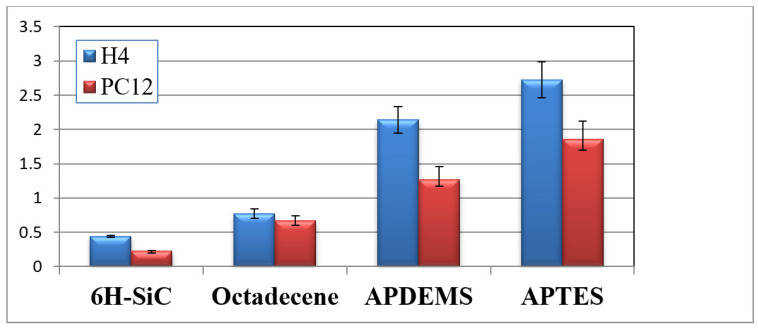
Proliferation of H4 and PC12 cell lines on (0001) 6H-SiC substrates vs. surface termination as determined by MTT assay analysis. The results are expressed as a sample distribution of the mean ($\bar{x}$) and standard error of the mean (σ_M_), and normalized to the PSt readings (*n* = 3) [19].

In principle, a biocompatible substrate promotes cell functionality and allows them to perform required chemical processes on the surface, such as specific signal transduction responses that lead to cell attachment and proliferation [9,10]. These processes are mediated by the interactions between cell-surface integrin receptors and proteins from the extra cellular matrix (ECM) that are adsorbed on the substrate. Moreover, cytoskeletal filaments are fundamental to the spatial organization of the cell. For example, actin filaments assemble into dynamic cell structures, such as lamellipodia and filopodia, which provide the cells a means of locomotion [11]. Cells tend to spread and increase their attached area when a substrate promotes the adhesion of suitable proteins [12]. Likewise, if a substrate is attractive to a cell, the cell filopodia and lamellipodia extensions couple strongly with the surface [13,14]. Consequently, cell morphology provides an indication of substrate biocompatibility. The combination of surface wettability, roughness, and charge plays an important role in the appropriate assembly of the structures [15,16,17]. As described above, the use of self-assembled monolayers (SAMs) to tune surface properties is particularly attractive for biotechnology and/or medical implant applications. SAMs can promote the immobilization of essential growth factors [18] and enhance cell proliferation [19], while maintaining close physical proximity and therefore electrical coupling between cells and the underlying substrate. SAM-based bio-functionalization of 6H-SiC was performed to assess its impact on cell morphology and substrate permissiveness. The SAMs were formed by reaction with aminopropyldiethoxymethylsilane (APDEMS) and aminopropyltriethoxysilane (APTES) using the silanization techniques described in [2] to create moderately hydrophilic surfaces. Quantification of cell proliferation was achieved using MTT assays which were performed in accordance [4] and shown in Figure 7.

#### 3.1.2. SiC Surface Functionalization to Detect Myoglobin

**Figure 8:**
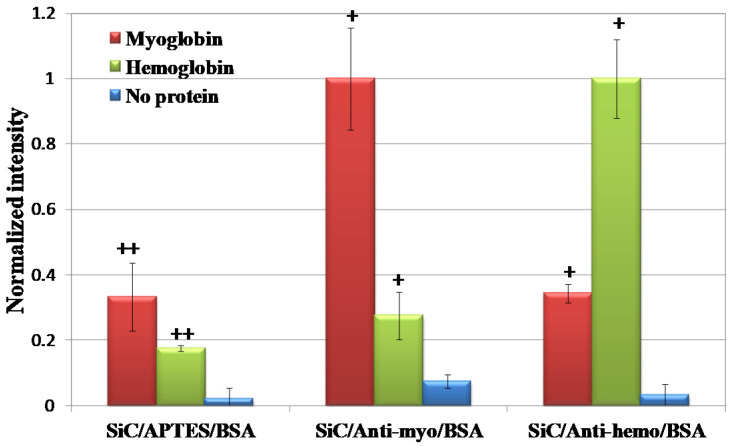
Normalized fluorescent intensity of Alexa-Fluor 488-labeled myoglobin and hemoglobin bound to APTES (**left**), anti-myoglobin (**center**), and anti-hemoglobin immobilized on SiC (**right**) with their respective controls. The results are expressed as the sample distribution of the mean ($\bar{x}$) and standard error of the mean (σM), and normalized to the positive control experiments in each test. + denotes *p*-values < 0.05 with respect to the antibody immobilized control and ++ denotes *p*-values < 0.05 with respect to the surface after APTES modification [19].

Myoglobin is released into the blood when heart or skeletal muscle is injured. In principle, the detection of myoglobin can alert medical professionals to a pending heart attack. Thus, the development of a sensor to detect its presence in low concentrations is clearly beneficial for patients with a high risk of death by myocardial infarction. Specificity of an immunosensor to an antigen of interest is one of the main properties that can be accomplished with immunosensors, as explained in [8]. For this reason, after performing characterization of anti-myoglobin immobilized on APTE-modified 3C-SiC, fluorescent microscopy was used to detect anti-myoglobin/myoglobin binding. The data is reported in Figure 8 as the sample distribution of the mean ($\bar{x}$; *n* = 9) and the standard error of the mean (σM). A *t*-test was performed for each surface with respect to their controls.

### 3.2. SiC Glucose Biosensor

SiC has long been used in advanced radio frequency systems due to its excellent carrier transport and high-power properties. Many of today’s point-of-use healthcare systems are hybrid implantable systems that combine radio frequency (WiFi and/or bluetooth) and biosensor technologies. Biosensors usually rely on relevant physiological parameters for continuous monitoring and an integrated antenna is often employed to send the received data to an external receiver. Therefore, implantable antennas have attracted considerable attention in recent years as a potential solution for communicating with implantable biosensors. Hybrid systems combining RF (antenna/wireless communication) and biosensor technologies are key to developing the next generation of continuous monitoring systems including implantable pacemakers and defibrillators, glucose monitors, insulin pumps, hearing aids, healthcare facility communication, medical and emergency equipment tracking, and remote patient monitoring, just to name a few.

A continuous glucose monitoring (CGM) sensor employing radio frequency (RF) signals was demonstrated using 4H-SiC [20]. The fabricated implantable RF antenna utilized semi-insulating SiC as the material of choice due to its great potential benefit as a robust bio and hemocompatible material for long-term in vivo systems. Distinguished by its innovation to utilize SiC as the device material, this system removes the need to encase this biomedical device inside biocompatible materials, thus addressing the short lifetime issue experienced by many contemporary sensors.

Unlike biosensors that require direct contact with interstitial fluids to trigger chemical reactions with functionalized surfaces, this SiC sensor does not require a direct interface to bodily fluids. The sensing mechanism is based upon shifts in the antenna resonant frequency as a function of changes in glucose levels electrically manifesting as corresponding shifts in blood permittivity and conductivity.

**Figure 9:**
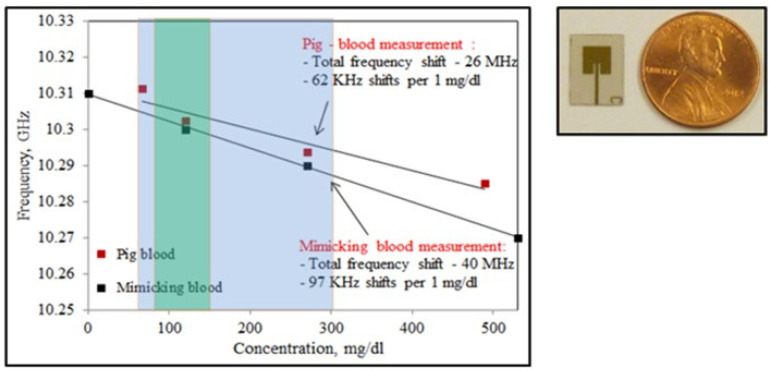
Measured relative resonance frequency shift dependence on glucose concentration. Photograph of the RF patch antenna on 4H-SiC. Note fairly linear response vs. glucose concentration [3].

From these results, it was observed that the antenna response to both a blood mimicking liquid and pig blood had similar trends in frequency shifts where the resonance frequency decreased with an increase of glucose levels. For a non-diabetic healthy person, the American Diabetes Association recommends a fasting plasma glucose level of 70–130 mg/dL, which is shown in Figure 9 (green shaded area). Below 60–65 mg/dL is known as hypoglycemia and above 240 mg/dL is known as hyperglycemia (shown in blue shaded areas). Note that the SiC-based sensor covers the normal-to-critical care regions with a reasonable slope in the frequency vs. glucose response at 10 GHz. These results demonstrate the potential use of biocompatible SiC to fabricate long-term, real-time continuous glucose monitoring of diabetic patients. Due to limitations in the Federal Communications Commission (FCC) broadcast bands, the sensor was redesigned to operate in the industrial, scientific, and medical (ISM; 2.45 GHz) band, and this work, while still on-going, has been quite promising and is discussed next.

**Figure 10:**
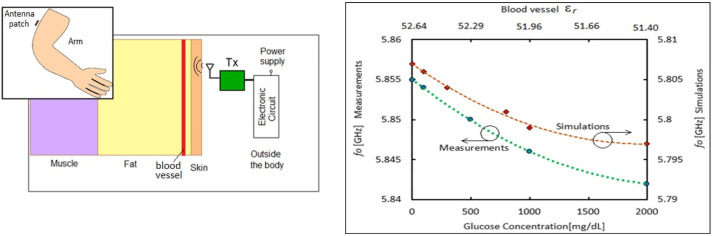
Non-invasive glucose sensing antenna configuration (**left**) on top of the arm to detect glucose changes in the cephalic vein (inset) along with HFSS™ simulation geometry. Measured vs. simulated data (**right**) using a tissue phantom, indicating that non-invasive glucose detection is possible with the remote antenna configuration [21].

Following the work of Afroz et al. [20], the USF SiC group continued this project with the specific objective of scaling the implantable sensor for the ISM band [21]. This non-invasive solution would be much more convenient for the patient, would not require surgery, and lower power WiFi communication would be allowed due to no signal attenuation in human tissue. Figure 10 depicts a glucose sensing antenna patch located above the upper arm with the antenna and external circuitry located outside the body.

Based on this data, it seems feasible to use a single RF sensing antenna to extract glucose information from blood non-invasively. What is needed now is the development of a sensor platform that can perform these measurements in real-time with an automated output of the assessed glucose value, which is the subject of future CGM work.

## 4. SiC Nanotechnology

Nanotechnology is a very broad field of exploration and technical development, which means different things to different people. In a broad sense, systems/objects on the nanometer scale are considered and one definition may be found in Wikipedia which states “Nanotechnology, also shortened to nanotech, is the use of matter on an atomic, molecular, and supramolecular scale for industrial purposes.” In the context of the present discussion, we present SiC nanotechnology using the classical definition with either three dimensions (nanoparticle), two dimensions (nanowire), or one dimension (nanowell) as the spatial feature(s) in the nanometer scale. Most of the reported works involving SiC nanotechnology use either nanoparticles or nanowires. Therefore, the following discussion relates to these types of nanostructures with their application to nanomedicine, in the present case, to treat cancer. For a more general discussion of SiC nanotechnology involving other applications, an excellent review article is available [22].

### 4.1. Nanowires and Nanoparticles

Nanotechnology enables innovative systems with unique properties and applications in several fields from sensors to nanomedicine [23]. For healthcare applications, the ability to tailor the material properties allows for the design of new nanosystems with enhanced performance for diagnostics, imaging, and oncotherapy [24,25]. Nanowires (NWs) based on cubic silicon carbide (3C-SiC) have a strong potential since they are chemically inert and compatible in the biological environment. Indeed, 3C-SiC has proved to be a bio and hemocompatible material, and some biomedical device prototypes have been successfully realized through thin-film SiC fabrication, as reviewed by Zorman et al. in [2]. The combination of 3C-SiC with silicon dioxide in core/shell NW structures (i.e., core/shell 3C-SiC/SiO_2_ NWs) opens more ways to engineer the surface via functionalization and decoration with macro-molecules and nanoparticles [26,27]. The potential use of core/shell NWs in nanomedicine is driven by the presence of the amorphous shell since it can modify the material behavior in the biological environment. Meanwhile, for blood-contacting applications, silicon oxide typically induces an aggregation and activation of platelets, promoting clot formation and acute inflammatory processes [28,29], as evidence from the 3C-SiC core/SiO_2_ shell NW system has not shown a deleterious impact on cells in vitro [30]. Furthermore, a peculiar feature of the core/shell NWs is their optical emission: the oxide shell enhances the core luminescence [31,32,33,34] when the nanosystem is excited by highly energetic sources, such as electron beams or X-rays. This property opens the possibility to exploit 3C-SiC/SiOx NWs as radiation-resistant scintillation nanostructures, which can be properly functionalized to play an active role for new oncotherapies, for example, such as X-ray excited photodynamic therapy (PDT) [31], in nanomedicine.

### 4.2. Photodynamic Therapy (PDT) to Treat Deep-Tissue Cancer Using SiC Nanowires

**Figure 11:**
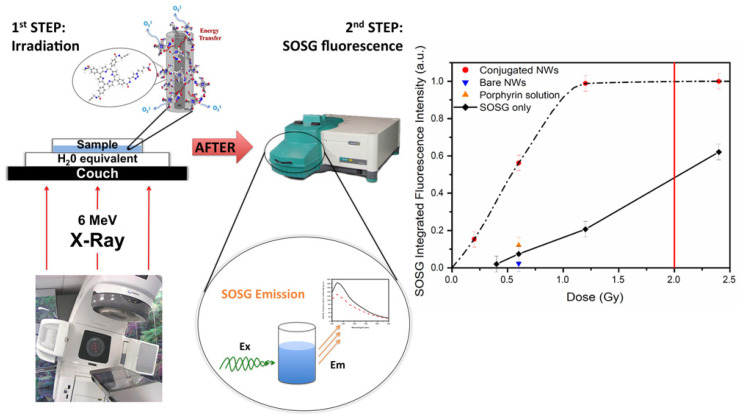
^1^O_2_ production excited by X-rays in a radiation therapy setup. Sketch of the experimental steps: a dish containing the sample solution is put on the couch and irradiated from the bottom, and then it is transferred to a spectrophotometer to acquire the fluorescence spectrum of the SOSG marker. The plot reports the measured integrated fluorescence intensity, proportional to ^1^O_2_ generated, as a function of the radiation dose. The experimental points are obtained from a SOSG kit in water, with H_2_TPACPP-functionalized NWs (red circles) or without NWs (black diamonds). The orange triangle at 0.6 Gy is the experimental point obtained from the SOSG kit in a water solution of mere porphyrin, while the blue triangle is obtained from the SOSG kit in a water suspension of NWs as grown. The usual dose for clinical treatment (2 Gy) is indicated by the red vertical line [2].

In the field of clinical oncotherapy, the standard protocols to control cancer growth and spread are still based on chemo and radio-therapy combined with surgical resection. New strategies, such as genetic approaches, are expected to deeply change cancer treatments in the near future. Alternative approaches aimed at killing tumors while minimizing side effects could be allowed by new classes of multifunctional nanomaterials and are presently under study. For instance, scintillation nanoparticles (NPs) were recently proposed in pilot studies [32,33] to activate Self-lighted Photodynamic Therapy (SLPDT) to treat deep-tissue cancer. SLPDT is a variation of well-studied Photo Dynamic Therapy (PDT) usually used to treat tumors on or just under the skin, or on the lining of internal organs or cavities through the generation of an active form of oxygen (singlet oxygen, ^1^O_2_) that destroys nearby cancer cells (definition from the US-National Cancer Institute [34]). In the above-mentioned pilot studies on SLPDT, photosensitizers are attached to the scintillation NPs and allow for light generation under X-ray irradiation. The photosensitizer (e.g., organic dyes, aromatic hydrocarbons, porphyrins, phthalocyanines, and related tetrapyrroles) [35] promotes, through singlet oxygen generation, oxidative stress to kill cancer cells. Since X-rays can penetrate body tissue, deep tumors can be reached and treated with this method (see Figure 11).

The work by Salviati et al. [2] provides an overview of the growth and optical properties, as well as some in vitro applications of core/shell 3C-SiC/SiO_2_ NWs and bare SiC NWs. It was shown, by the analysis of cell proliferation, cell cycle progression, and oxidative stress, that the core/shell NWs are cyto-compatible over a time of up to 10 days. They are effectively internalized by cells through the macropinocytosis mechanism (phagocytosis only in the THP-1 model) and sporadically by direct penetration. For all the cell lines studied, the intracellular presence of NWs induced the same molecular events: peroxidation of membrane lipids and oxidation of proteins. These effects are late-stage and may be limited by the activation of protection systems; for instance the effect of ROS is not acute and effectively countered by the intracellular scavenger. These results highlight that core/shell 3C-SiC/SiO_2_ NWs do not elicit either mid-term (72 h) or long-term (10 days) cytotoxic activity leading to irreversible cellular damages or death.

### 4.3. Near-Infrared Photo-Immune Therapy (NIR-PIT) Using SiC Nanostructures

While singlet oxygen generation does kill cancer cells, it also kills healthy cells within close proximity. An alternate approach, proposed by a research team led by S. E. Saddow and colleagues from Italy, Hungary, and the NCI (Bethesda, MD), is seeking to use SiC nanostructures to facilitate cancer treatment via photo-immune therapy (PIT). This research is ongoing and details may be found on the National Institutes of Health Grantome website [35]. The concept in the work by Saddow et Al. is to enable ‘deep-tissue’ cancer treatment by using X-ray irradiation to stimulate 690 nm NIR (near-IR) emission from SiC nanostructures to activate the PIT process using antibody conjugates containing the IR-700 molecule. This research is specifically aimed at treating patients with such pernicious cancers as liver, pancreas, etc. So far, a very promising SiC-based coreshell nanostructure system (ZGC:SiC) has been developed and reported by Beke et al. from this project [36].

### 4.4. Room-Temperature DNA Assays Using a-SiC-Coated Si Nanowires

Semiconducting nanowires are of a growing interest for nanoelectronic devices, in particular, for sensing applications [37]. While silicon is the most widely used material in this field, it lacks long-term stability in aqueous solution [38,39] and therefore is not suited for most liquid environments. Indeed, chemical resistance to the medium is essential for device reliability and signal stability. SiC appears as a promising material for these applications [40] thanks to its semiconducting properties as well as its very high chemical stability and biocompatibility [41]. However, SiC is much more difficult to etch due to its high physical and chemical stability compared to Si material. Thus, coreshell Si/SiC nanowires could be an advantageous compromise between these two approaches [42]. Applications include ion-sensitive FETs (ISFETs), chemical FETs (ChemFETs), and other related sensing devices. These devices would benefit from the strong chemical and physical stability of the SiC shell and from the superior electron transport within the Si core.

**Figure 12:**
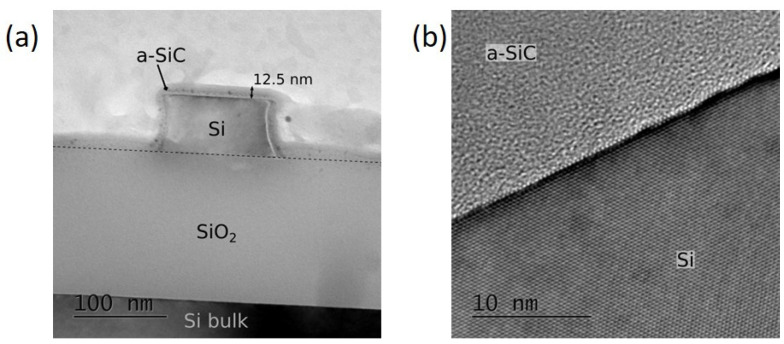
Si/SiC nanowire DNA sensor structure. (**a**) TEM cross-section image of a Si NW etched from SOI substrate and covered with *a*-SiC deposited by PECVD, and (**b**) HR-TEM image at the Si/*a*-SiC interface [43].

In [43], TEM analysis was conducted to investigate the structural and crystallographic differences between the two methods. Figure 12 depicts the structure after Si NW etching and *a*-SiC coating via PECVD. Si NWs exhibited a rectangular shape after dry-etching of the patterned topmost Si film and were then coated with a thin layer of *a*-SiC. Despite the low contrast between *a*-SiC, SiO_2_, and the glue matrix, TEM images show that the *a*-SiC coating is conformal and covers the entire exposed Si and SiO_2_ surfaces with a thickness of about 12.5 nm, decreasing to 10 nm on the NW side walls. Since *a*-SiC behaves like an insulator, devices remain electrically isolated from one another despite the SiC layer covering the SiO_2_ surface. The high-resolution image (Figure 12b) shows no voids or defects at the Si/*a*-SiC interface and low stress on the first five Si atomic layers.

**Figure 13:**
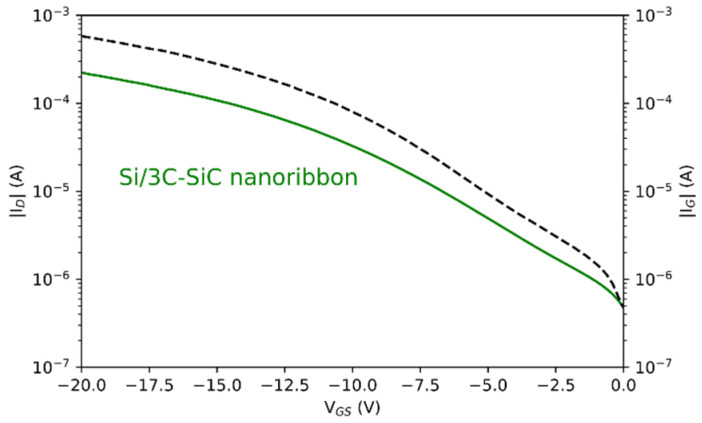
Transfer characteristics of backgated core-shell Si/*a*-SiC nanoribbon devices: *a*-SiC-coated SOI device resulting in thin SiC shell. Drain current plotted with solid line and gate current with dashed line [43].

Devices were characterized electrically using current-voltage measurements after defining metal contacts. Typical *I*_D_-*V*_G_ transfer characteristics are shown in Figure 13 and is plotted vs. gate current *I*_G_. Electrical characterization was conducted on nanoribbons (NRs) only because they provided higher reproducibility than NWs. The devices were backgated from the Si substrate and biased with a drain voltage of *V*_D_ = 4 V.

In this study, the plasma-enhanced chemical vapor deposition (PECVD) of a passivating *a*-SiC layer allowed for the development of potentially high-durability coreshell Si/*a*-SiC devices designed to be operated in liquid environments. Based on these results, further research will focus on coreshell Si/*a*-SiC FETs using the PECVD of *a*-SiC, hence benefitting from the reproducible electrical performances exhibited throughout the process.

## 5. SiC Implants

Implants refer to the general class of devices/materials that are surgically inserted into the human body. They typically involve dental prostheses such as artificial teeth, cardiovascular stents to prevent vascular collapse after angioplasty, or biosensors such as those used in modern glucose monitoring systems. There are also skeletal (bone) implants traditionally made with metals for such common applications as artificial knees and the like and have a limited lifetime [44]. In the case of bone implants, they are often treated to display a ceramic surface to tissue to reduce and/or eliminate irritation and thus reduce body rejection. Perhaps the most interesting application of SiC implants involves the human nervous system, namely either the central (brain) or peripheral (limbs) system. In this section, we will review some excellent examples of the use of SiC both in bone and to provide a bi-directional signal pathway to the nervous system.

### 5.1. Bone Scaffolds

Polycrystalline silicon carbide (SiC) has been extensively used in the ceramics industry and is mainly known for its mechanical strength and refractory characteristics. Hence, it is used extensively as a grinding agent as well as a high-strength machine tool coating. As examined previously, SiC is highly biocompatible and, when implanted, has demonstrated minimal inflammatory or negative tissue response [2,3]. In an effort to explore the potential of SiC as a substrate for bone growth, nanoporous SiC (np-SiC) was mineralized with sol-gel coatings of hydroxyapatite (HA) under various deposition conditions, as described in [2].

HA is amongst the most ubiquitous phases of calcium phosphate-based materials and has been widely used as a bone substitute material for orthopedic and dental applications due to the biological and chemical similarity between HA and the mineralized bone matrix of human tissue [45,46,47]. The ability of HA to form a chemical bond with host bone tissue renders HA very advantageous as a synthetic bone substitute for clinical applications. The osteoconductive characteristics of HA also makes it more popular compared to other ceramic or metal-based bone substitutes. Furthermore, HA is biocompatible, making it a preferred synthetic material when compared to other existing bone implant systems, such as allografts and xenografts [45,46,47]. HA has been synthesized in bulk form from powders and sintered ceramics, as well as in thin-film coatings on metal and ceramic substrates using a variety of solid-state, chemical, and vapor-phase techniques.

**Figure 14:**
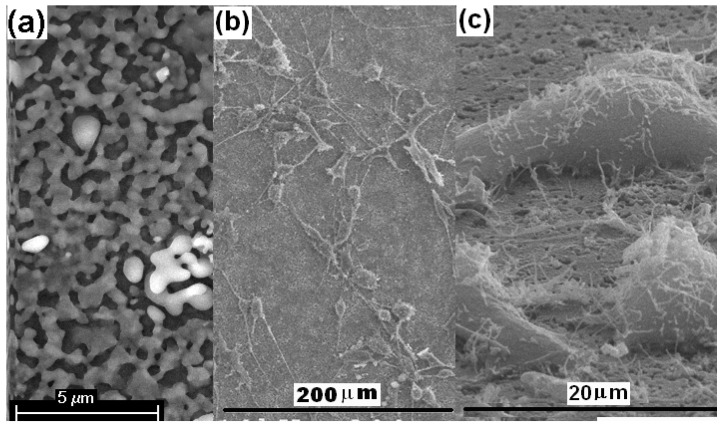
SEM micrograph (**a**) of 1.4 µm of HA film on ~16 nm np-SiC obtained after thermal treatment at ~900 °C for 12 h. (**b**) MG-63 cell attachment to 1.4 µm thick HA on ~16 nm np-SiC at lower magnification and (**c**) higher magnification [2].

In [2], HA films of varying thickness (0.7–4 µm) were synthesized on nanoporous SiC comprised of nanometer-sized pores (~10 nm, ~16 nm, and ~50 nm) using a sol-gel method employing phosphorus pentoxide (P_2_O_5_) and calcium nitrate (Ca(NO_3_)_2_, 4H_2_O) as precursors. The formation of nanostructured HA with grain sizes close to the dimension of the pores on the underlying substrate has been identified up to the calcination temperature of ~500 °C in air (Figure 14). A smooth, dense, and crack-free HA film with low porosity has been obtained after sintering the nanostructured HA film at ~900 °C in air. The HA film of ~1.4 µm thickness showed good cohesive strength between the particles. The cohesive strength, however, decreased with an increase in the film thickness above ~2 µm. Cell attachment studies revealed that for a ~1.4 µm thick HA film, good adhesion with both ~16 and ~50 nm np-SiC was exhibited throughout the cell culture due to the adhesive strength of the HA film with substrates of increasing pore size [2].

### 5.2. Dental and Orthopedic Implants

**Figure 15:**
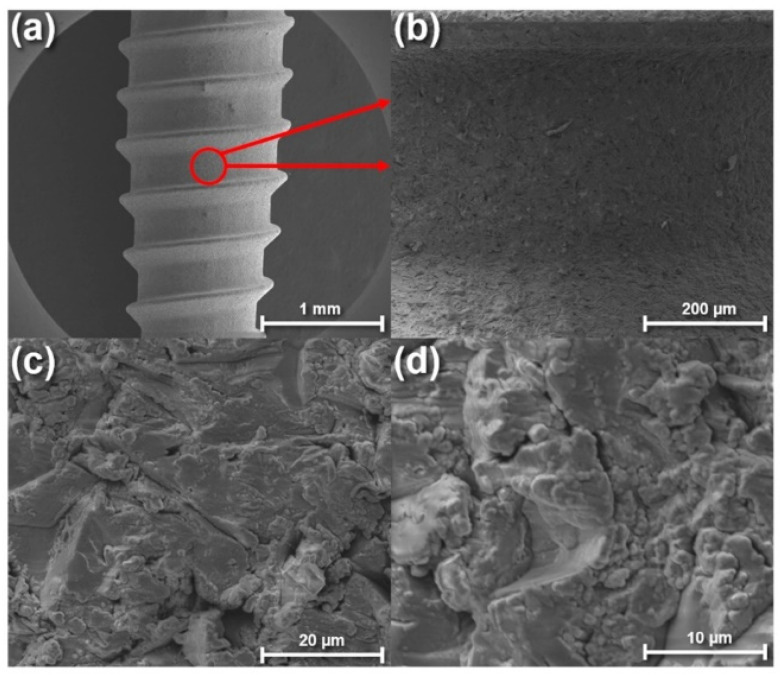
Scanning electron microscope images of a SiC-coated titanium implant at various magnifications. Image (**a**) shows the overall surface of the implant, whereas images (**b**–**d**) show detailed images of the implant surface morphology at increasing magnifications [47].

Dental implants are perhaps one of the most common devices implanted in the human body for long-term use. There are several issues mostly relating to the osseointegration and biocompatibility of the implant to tissue (soft and bone). Some examples of superior implants using SiC are shown below (Figure 15) with details of a SiC-coated dental implant reported in [47].

**Figure 16:**
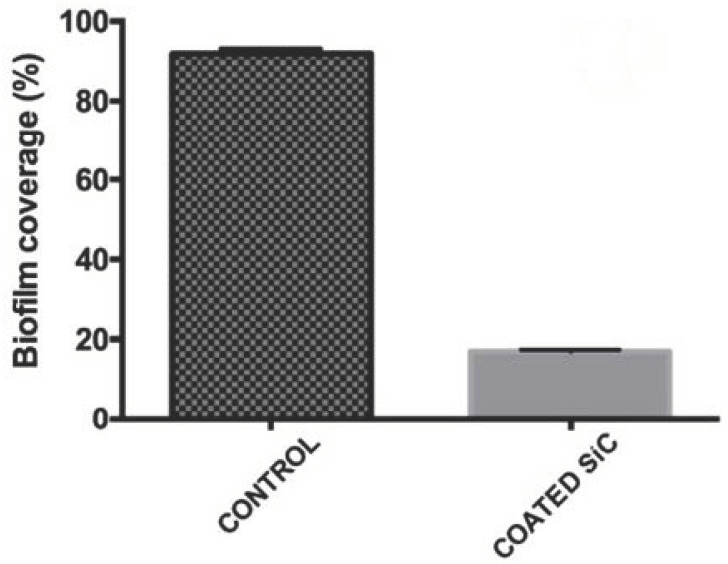
Live coverage of Streptococcus mutans and Streptococcus sanguinis after 24 h of culture on the non-coated (control) and SiC-coated surfaces [48].

One of the important observations of using SiC for dental implants is the natural bio-fouling resistance of this material, which is discussed in detail by Franca et al. in [1]. Biofilms are inhibited when SiC surfaces are presented to biological matter as shown in Figure 16 [48]. This property, along with the structural properties of SiC, make this an ideal material for dental implant applications.

### 5.3. SiC Neural Implants

The potential impact of permanent neuro-compatible implantable devices to assist millions who experienced brain and spinal cord injury and/or limb loss is tremendous in both restoring patient functionality as well as quality of life. Until now, no known reliable solution to this challenge has been found, with most of the current technology relying on materials not compatible with the neural environment, such as inorganic materials like silicon, tungsten, or platinum to polymer-based insulators such as parylene-C. SiC and, in particular, 3C-SiC appears to offer the material properties which would meet this challenging application: the evidence of bio and hemocompatibility; tailorable doping profiles for the seamless integration of electronics; high material durability within harsh, corrosive environments; and, lastly, excellent thermal conductivity [1].

**Figure 17:**
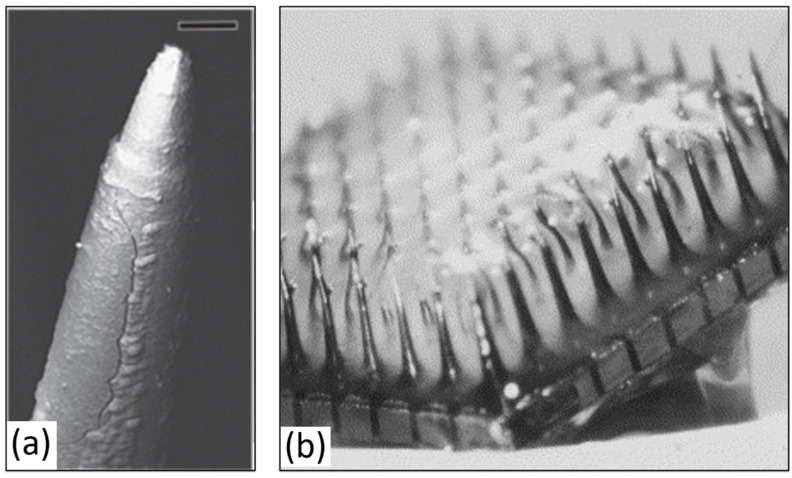
In vivo failures common in today’s heterogeneously integrated silicon-based neural probes. The Utah intracortical electrode array (Blackrock). (**a**) Side view of an electrode with a large longitudinal crack in the parylene-C insulation adjacent to surface irregularities (20 µm scale). (**b**) Explanted UIEA array at 10 months. The array was completely encapsulated. Reproduced with permission from Bernardin ©2018 [50].

The only neural implant approved for limited human use to date is based on the University of Utah intracortical array [49]. Due to numerous challenges, long-term reliable performance has yet to be achieved, some of which has been attributed to the heterogeneous material fabrication consisting of the silicon shank, platinum/iridium-based electrode conductors, and parylene-C insulation. Figure 17 shows one example of device failure observed in vivo which is representative of typical implant issues encountered with Si-based implants. 

**Figure 18:**
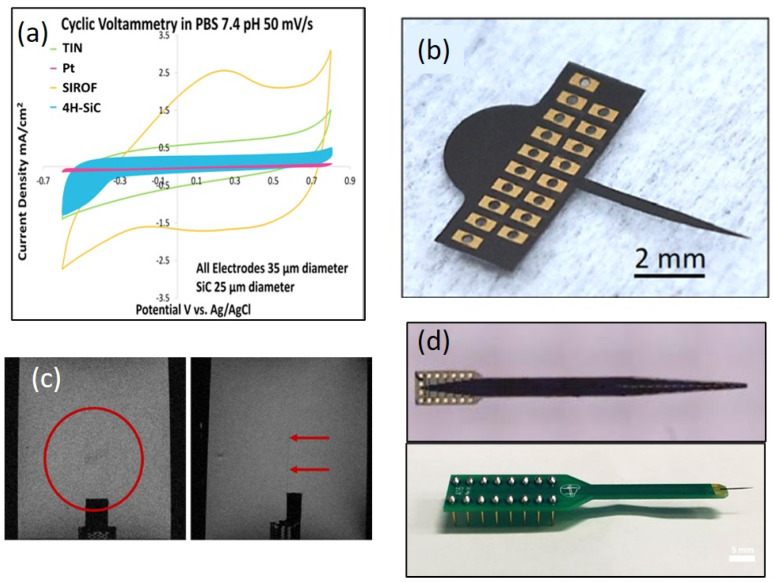
Examples of SiC INI devices developed in the USF SiC group. (**a**) CV performance comparison of an all-SiC (4H-SiC polytype) MEA showing superior performance to Pt electrodes of the same form-fit [51]. (**b**) Free-standing all-SiC (3C-SiC on SOI) 16-channel functional INI used to assess the MRI compatibility [54]. (**c**) Sagittal (left) and coronal (right) MRI images @7T showing no image artifacts and nearly transparent performance of 3C-SiC in a brain-tissue phantom. (**d**) Photographs of a released C-based *a*-SiC INI device ~1 µm thick (top) and a packaged device (bottom) with Si-backing for structural support mounted on a NeuroNexus package header for in vivo testing [53].

Fortunately, numerous instances of SiC being used as a robust material for implantable neural implant (INI) applications have been reported [50,51,52,53,54]. In the USF SiC group, we have been working on this challenge for nearly two decades and have developed several possible solutions to address long-term in vivo INI issues: an all-SiC monolithic MEA constructed using 4H-SiC [51], an alternate device based on 3C-SiC on either bulk Si or SOI substrates [52], and, most recently, an ultra-thin interface using *a*-SiC as the base and capping insulation, which sandwiches a carbon electrode created using pyrolyzed photo-resistant film (PPF) [53]. Not only has excellent electrical performance been demonstrated in PBS, but in the case of the 3C-SiC all-SiC interface on SOI, excellent MRI compatibility has been observed in a 7T animal-bore MRI system at the Moffitt Cancer Center [54]. Details of this research is included in [1]. A preview of these results is shown in Figure 18.

A comparison of both the all-SiC and C-based SiC electrodes has been complied by C. Feng and is shown in Table 1.

## 6. Summary

In this paper, an introduction to SiC as a robust and highly useful material for biomedical devices and applications has been presented. Starting with a review of in vitro and in vivo SiC performance, the discussion moved to SiC surface modification through the use of self-assembled monolayers (SAMs) and their use in the biosensing of myoglobin, as well as to the alteration of the surface biocompatibility and wettability. The use of semi-insulating 4H-SiC for a 10 GHz RF patch antenna with the ability to measure glucose concentration in vitro was presented, followed by the development of a non-invasive glucose sensor in the ISM band. The development of SiC nanostructures, namely nanowires for photodynamic therapy (PDT) to treat deep-tissue cancer via the generation of singlet oxygen (^1^O_2_) via X-ray excitation, was presented. This was followed by an introduction of the use of ZGC:SiC nanoparticles under development to treat deep-tissue cancer via excitation of near-IR (NIR) light to activate a photo-immune therapy (PIT) for cancer therapy. Having completed the SiC materials portion of the paper, SiC devices, ranging from bone scaffolds to dental implants, were presented with the final device topic being neural implants (INI). The development of three SiC-based INI devices was presented and the MRI compatibility of an all-SiC INI comprised of 3C-SiC on SOI showing nearly transparent performance in a 7T animal-bore MRI tool was presented. Based on this extensive body of work, SiC is poised to enable a new generation of ‘smart’ biomedical devices for human healthcare applications, as the semiconducting nature of SiC allows for the integration of electronics on the implanted device platform directly, from signal stimulation and recording electronics to WiFi communication and complex waveform analysis. The future of SiC as a smart biomedical device platform appears to be bright and continuing technological development worldwide should bring numerous novel, long-term devices to the marketplace. The work presented here in a compact ‘review style’ form may be found in a comprehensive book series starting with the first edition that focused mainly on the material properties of SiC [2]. The second edition focuses mainly on preliminary biomedical device prototypes [3], while *Silicon Carbide Technology for Advanced Human Healthcare Applications* [1], the final book in the series, presents the research outlined in this paper, along with additional work on SiC neuron interaction with SiC and graphene, *a*-SiC-coated retinal implants, and graphene-based biosensors. The future of SiC as a material of choice for the design of advanced biomedical devices appears to be very bright and, while a market analysis has yet to be done, the global medical device market size was USD 432.23 billion in 2020 and is projected to grow to USD 657.98 billion in 2028 at a CAGR of 5.4% in the 2021–2028 period [59]. One can speculate that SiC biomedical technology will play an important role in this global market as the long-term reliability of this class of devices is unparalleled. Time will tell but the hope is that this paper will have motivated the next generation of biomedical technologists to consider the development and adoption of SiC for next-generation medical devices.

## Figures and Tables

**Table 1:** Electrochemical properties of SiC and common mINP electrode materials.

Material	Recording Area (kµm^2^)	Impedance @1kHz (kΩ)	Charge Storage Capacity (mC/cm^2^)
3C-SiC	0.49	71.6	205
PPF/*a*-SiC	1.9	24.8	14,160
Pt	1.9	103.8	12
PEDOT/CNT [55]	2.83	15.0	6
Carbon-nanotube fiber [56]	1.450	14.1	372
Graphene fiber [57]	0.749	37.9	798
IrO_x_ [58]	0.177	132.9	29
TiN [55]	2.83	54.8	5
